# A pharmaco-metabolomics study of *Glycyrrhiza glabra, Boswellia sarca*, and *Acacia nilotica* in acute allergic dermatitis

**DOI:** 10.1007/s10787-025-01761-7

**Published:** 2025-05-19

**Authors:** Bassant M. M. Ibrahim, A. F. Yousuf, M. M. El-Shawwa, Mona A. Mohammed

**Affiliations:** 1 Department of Pharmacology, Medical Research and Clinical Studies Institute, National Research Centre, Dokki, Giza, 12622 Egypt; 2 Department of Physiology, Faculty of Medicine for Girls, Al-Azhar University, Cairo, Egypt; 3 Medicinal and Aromatic Plants Research Department, Pharmaceutical Industries Research Institute, National Research Centre, Giza, Egypt

**Keywords:** *Acacia nilotica*, *Boswellia sarca*, *Glycyrrhiza glabra*, Acute dermatitis, Histamine

## Abstract

Acute allergic contact dermatitis is an inflammatory skin condition characterized by swollen, itchy lesions. This study aimed to evaluate the soothing and wound-healing effects of fixed and volatile oils of *Boswellia sarca*, as well as extracts of *Glycyrrhiza glabra* and *Acacia nilotica*, on acute contact dermatitis in rats. Phytochemical analysis revealed the presence of flavonoids, tannins, saponins, triterpenoids, alkaloids, and cardiac glycosides in *Acacia nilotica* and, *Glycyrrhiza glabra* extracts, with *Boswellia sarca* showing a dominance of volatile oils. The study included a normal group and six acute allergic dermatitis groups induced by subcutaneous histamine injection. One group served as a positive control without treatment, while five groups were treated topically at inflamed sites with *Boswellia sarca* oils, *Glycyrrhiza glabra*, and *Acacia nilotica* extracts, alongside betamethasone as a standard treatment. The effects were evaluated through inspection, serum levels of ICAM-1, LTB4, and ILβ-4, as well as histopathological and immunohistochemical analyses. GC/MS analysis identified Incensole acetate (50.12%) and Incensole (32.44%) as major compounds in *BS* fixed oil, with significant terpenoids and volatile components. Metabolomic profiling using LC-MS/MS highlighted diverse secondary metabolites in *Acacia nilotica* and, *Glycyrrhiza glabra*, including polyphenolic acids, flavonoids, and amino acids, showcasing their therapeutic potential. All topical treatments reduced ICAM-1 and LTB4 levels to varying degrees and exhibited better histopathological and immunohistochemical results compared to the untreated positive control group. Among the treatments, *Boswellia* oils and, *Glycyrrhiza glabra* extracts demonstrated the most effective soothing and curative effects on allergic dermatitis. *Boswellia sarca* oils and, *Glycyrrhiza glabra* extract showed the best soothing and curative effects against allergic dermatitis.

## Introduction

Acute allergic contact dermatitis is an inflammatory disease characterized by swollen itchy skin lesions, that occur as a result of contact with a chemical (Ali et al. 2024). The pathogenesis of dermatitis may include disrupted epidermal barrier function, immune-dysregulation, and IgE-mediated sensitization to food and environmental allergens (Zheng et al. 2011). One of the most potent mediators of inflammation is histamine which causes allergic and inflammatory reactions in the form of vasodilation and oedema in addition to high chemoattractant activity. Histamine binds to eosinophil H4R leading to increased expression of intracellular adhesion molecule-1 (ICAM-1), which enhances eosinophil migration to the inflammation region, together with promotion of rearrangement of actin filament (Branco et al. 2018). In addition to emollients and topical hydrating agents (Ali et al. 2024), topical corticosteroids are the drugs of choice for treatment of allergic dermatitis during flare ups, this owes to their immunomodulatory anti-inflammatory activity such as betamethasone, which acts as an anti-inflammatory, anti-allergic and immunosuppressive medication. Despite their common use in treatment of dermatitis, corticosteroids have the risk of triggering hypersensitivity due to their low molecular weight and high lipophilicity which enhance skin penetration and sensitization of T lymphocytes with subsequent development of allergic contact dermatitis (Schimmer and Parker 1996). Therefore, natural products as *Boswellia sarca* volatile and fixed oils, extract of *Glycyrrhiza glabra* (Licorice) and *Acacia nilotica* act as a potentially safer alternative to topical steroids, which can be used in cases of inflammation associated with allergy due to their anti-inflammatory and anti-allergic effects (Ibrahim et al. 2016; Leite et al. 2022; Rauf et al. 2024). *Boswellia*, recognized for the aromatic resin derived from its trees, possesses diverse pharmacological applications, particularly as agents with anti-inflammatory actions (Taleb et al. 2024). In addition, the resin’s components, such as boswellic acids, have exhibited encouraging outcomes in the management of allergic conditions due to its anti-leukotriene activity (Ibrahim et al. 2016). Licorice is extensively incorporated in clinical formulations. The key bioactive compounds found in licorice comprise triterpenes, flavonoids, and polysaccharides, which exhibit anti-inflammatory and immune-regulatory properties. *Acacia nilotica* demonstrated inhibitory effects on carrageenan-induced paw edema and yeast-induced pyrexia in rats. Furthermore, it elicited a notable enhancement in the hot plate reaction time in mice.

The evolution of separation and characterization technologies has provided essential means to identify the active constituents of medicinal plants and specifically isolate the biologically active compounds, thereby making the utilization of the entire plant as a therapeutic drug for treating specific diseases no longer acceptable (Aboul Naser et al. 2024; Ellaithy et al. 2022; Mohammed et al. 2024a). Metabolomics stands as the latest and well-integrated discipline that contributes significantly to this domain. The field of metabolomics has rapidly advanced and demonstrated substantial influence on both foundational and applied sciences. The concept of a metabolome was introduced in 1998 by Oliver et al., defining it as the comprehensive collection of low molecular weight compounds within a cell that are necessary for its sustenance, growth, and regular functions, contributing to the metabolic processes of a cell at a given physiological or developmental stage. Since metabolites are products downstream of gene transcription and translation (proteins), metabolomics methodologies can offer a clearer insight into the phenotype of a biological system. However, compared to the genome and proteome, the metabolome is notably intricate; for example, the entire plant kingdom is estimated to harbor 200,000 or more metabolites and phytochemicals, with the human metabolome database currently containing 41,815 metabolite entries (Mohammed et al. 2024b, 2022a). The distinctive physicochemical characteristics of various categories of metabolites contribute significantly to the intricate nature of metabolomics investigations, which have been pivotal in driving the advancement of diverse methodologies and the utilization of a broad array of analytical platforms. Multiple analytical platforms must be employed in a supplementary fashion to capture the extensive chemical variability. The predominant technologies for metabolite identification, commonly known as “work-horses,” involve mass spectrometry (MS). To improve resolution, chromatographic separation methods such as liquid chromatography (LC) or gas chromatography (GC) can be combined with MS depending on the complexity of the samples.

The present study was designed to investigate the soothing anti-inflammatory and anti-allergic effects of volatile and fixed oils of *Boswellia*, as well as extracts of *Licorice* and *Acacia nilotica* on acute allergic dermatitis due to chemical irritation induced in rats by subcutaneous injection of histamine.

## Materials and methods

### Phytochemical study

#### Extraction, determination, identification and analysis of three plants

The three plant species *Glycyrrhiza glabra* (GG), *Boswellia sarca* (BS), and *Acacia nilotica* (AN) were sourced from the Haraz market in Egypt. The leaves and fruits of these plants were dried, and subsequently ground into powder. A total of 500 kg of powdered leaves or fruits from the three plants underwent extraction using ethanol (70%) for GG and AN, while BS was extracted using petroleum ether 40–60 through a soaking process at ambient temperature. Following extraction, the combined alcoholic extracts were concentrated under reduced pressure at 45 °C utilizing a rotary evaporator. This process resulted in the production of 70 g, 30 g, and 66 g of residue from GG, BS, and AN respectively (Mohammed et al. 2020, 2022b).

### Chromatographic spectroscopy section

#### Phytochemical screening of three plant extracts.

The identification of various compounds in the extracts was carried out using specific reagents and established methods. A-naphthol sulphuric acid reagent, as described by Lewis and Smith, was used for compound detection(Stephen 1977). The presence of tannins was determined using Shellard’s method (Shellard 1957). To detect alkaloids, 1 mL of the alcoholic extract filtrate was mixed with 2 mL of Dragendoff’s reagent, resulting in the development of a turbid orange color, which signified the presence of alkaloids. This result was further confirmed using Mayer’s reagent**,** where the formation of a yellow precipitate indicated the presence of alkaloids(Harborne and Harborne 1973). The presence of flavonoids was suggested by the appearance of a yellow color, as noted by Trease and Evans (1989). Additionally, when magnesium and HCl were added to a separate portion of the ethanolic extract, the formation of a red color indicated the presence of flavanones and/or flavonols (Shinoda 1928). The potential presence of saponins was inferred from the observation of a persistent froth lasting approximately 30 min (Shellard 1957). Lastly, the detection of steroids and triterpenoids was based on the appearance of a green color in the upper layer and a deep red color in the lower layer, respectively, as per Hanson’s method (Hanson 1972). 

#### Determination of volatile oil content

The fruits of *Boswellia sarca* sourced from Egyptian markets were utilized for the quantification of volatile oil content. Extraction of the volatile oil from each dried resin specimen was carried out using the water distillation method for duration of 4 h in a Clevenger’s apparatus as described by Guenther (Guenther et al. 1959). The resulting essential oil from each treatment underwent dehydration individually with anhydrous sodium sulfate and was stored in a deep freezer until subjected to GC/MS analysis. Each sample was subjected to triplicate analysis, and the average values of the oil content percentage were documented (Rhimi et al. 2022). 

#### Sample preparation GC–MS identification of the chemical composition of volatile oils

The specimen was dissolved in chloroform and analyzed using a Gas Chromatography-Mass Spectrometry (GC–MS) system. This system, an Agilent Technologies 7890B GC coupled with a 5977 A mass spectrometer, was located at the Central Laboratories Network of the National Research Centre in Cairo, Egypt. Chromatographic separation was achieved on HP-5MS column (30 m × 0.25 mm, 0.25 μm film thickness) using helium as the carrier gas (3.0 mL/min). The temperature program started at 40 °C for 1 min, ramped to 200 °C at 10 °C/min, held for 1 min, then to 220 °C at 20 °C/min, held for 1 min, and finally to 320 °C at 30 °C/min, held for 3 min. The injector and detector temperatures were 250 °C and 320 °C, respectively. Electron ionization (EI) at 70 eV was used for mass spectral acquisition (m/z 30–550), with a solvent delay of 2.5 min. Mass spectrometer conditions were: mass temperature 230 °C, quad temperature 150 °C. Component identification was performed by comparing the obtained spectra with those in the Wiley and NIST Mass Spectral Libraries (Taleb et al. 2024).

#### Determination of total lipid concentration.

Five hundred grams of air-dried *Boswellia sarca* fruit powder underwent continuous extraction using petroleum ether (40–60 °C) in a Soxhlet apparatus until complete extraction was achieved. The solvent was subsequently removed using a rotary evaporator at 40 °C to dryness. The resulting extract was then placed in a vacuum desiccator until it reached a constant weight (Ibrahim et al. 2024).

#### Preparation of fatty acid methyl esters (FAME)

Fatty acid methyl esters are generated through a reaction catalyzed by alkali, involving fats and methanol, with the addition of 2 M potassium hydroxide, and subsequently introduced into hexane (Mohammed et al. 2021).

#### Identification and quantitative determination of fatty acids by GC/MS

A GC–MS system (Agilent Technologies 7890B GC/5977 A MS) at the Central Labs Network, National Research Centre, Cairo, Egypt, was used. The GC was equipped with an HP-5MS column (30 m × 0.25 mm i.d., 0.25 μm film). Analyses were performed using He as carrier gas (2.0 mL/min) in splitless mode with 1 μL injection. The temperature program was: 50 °C (hold 5 min), ramp 5 °C/min to 100 °C (hold 0 min), ramp 10 °C/min to 320 °C (hold 10 min). Injector/detector temps: 280/320 °C. EI (70 eV) mass spectra (m/z 25–700) were acquired with a 4 min solvent delay. Mass temp: 230 °C, Quad: 150 °C. Compound identification was based on spectral matching with Wiley and NIST libraries (Mohammed et al. 2021). 

#### Chromatographic conditions UPLC-HRMS

HPLC: Gradient elution employed a mobile phase of H2O/0.1% formic acid (A) and acetonitrile (B) at 200 μL/min. The gradient: 5% B (1 min), linear to 30% B over 20 min, linear to 98% B over 27 min (hold 3 min), linear to 5% B over 1 min. Detection: 280, 330, and 254 nm (El-Gengaihi et al. 2020; Mohammed et al. 2022a). UPLC-HRMS: Mobile phase flow rate: 400 μL/min. Gradient: 5% B (linear to 20% B in 5 min), linear to 98% B in 8 min (hold 1 min), linear to 5% B in 1 min (Mohammed et al. 2022a). MS: AIF MS scan (resolution: 70,000), AGC target: 1e6 ions, max IT: 50 ms, m/z 100–1200, microscans: 1. HCD fragmentation at NCE 15.0 eV (*z* = 1) in the collision cell. (Metabolomics lab, Institute of Plant Genetics, Poznan, Poland)(Mohammed et al. 2021).

### In vivo pharmacological study

#### Materials

##### Animals

Forty three male Albino Wistar rats (150–175 g body weight), had been obtained from the animal house colony of the National Research Centre, Dokki, Giza, Egypt, and were housed in sterilized stainless-steel cages in optimum temperature (23 ± 1 °C) and artificial illumination (12 h dark/light cycle). All rats were fed standard laboratory diet, and were allowed free access to water.

*Guidelines of animal ethics*: Animal procedures followed the regulations of the “Ethics Committee of the National Research Centre” and the recommendations of the “Institutional Animal Ethical Committee (IAEC) and the National Regulations of Animal Welfare”, additionally, the results were reported in line with “Animal Research: In-Vivo Experiment Reporting (ARRIVE)”.

The experimental ethics approval of the “Ethics Committee of the National Research Centre” was acquired under the number 19/209.

##### Chemicals and drugs

- Histamine, Formaldehyde and Diethyl ether were purchased from “Sigma Aldrich company.” (USA).
- Elisa kits for evaluation of intracellular adhesion molecule 1(ICAM-1), leukotriene B4 (LTB4) and interleukinβ 4 (ILβ4) levels were purchased from Elabscience (USA).
- betamethasone (*Betaderm*^®^) cream used as reference treatment, was purchased from the “Egyptian International Pharmaceutical Industries company” (EIPICO) in Egypt. Every gram of *Betaderm*^®^ contains betamethasone as betamethasone 17-valerate 0.05% in an aqueous base.

#### Methods

##### Induction of dermatitis

A pilot study was performed prior to selection of the most suitable dose of histamine which causes local allergic and inflammatory reactions. Eight rats had their dorsal hair shaved, then were subcutaneously injected with histamine in increasing doses starting from 5 µg up-till 40 µg. After injection of each dose every rat was observed for 30 min for the onset of redness or itching. It was observed that the 40 µg dose produced the fastest onset and highest degree of itching. Accordingly, 40 µg subcutaneous injection of histamine was selected for induction of chemical dermatitis in the present study.

##### Study design

The dorsal aspects of all rats were shaved then the animals were divided into seven groups (*n* = 5 per group), as follows:

Normal group: for which hair shaving only was done.

Six groups were subcutaneously injected with 40 µg of histamine. And were classified as follows: Positive control group: was subcutaneously injected with 40 µg of histamine and did not receive treatment, reference group treated with betamethasone cream, volatile oil of BS group, fixed oil of BS group, extract of GG group, and extract of AN group. All treated groups were subcutaneously injected with 40 µg of histamine and after that by 1 h, thin films of treatment were applied to the animals’ skins at the sites of redness and scratches in all groups. Each 1 ml viscid solution contains 5 mg of the extract.

##### Inspection and grading of allergic reactions

Skin redness and pruritus were observed throughout the experiment every 30 min for 180 min and graded as none (no skin changes), mild (faint redness), moderate (deep redness or superficial scratches or both), severe (deep scratches).

##### Preparation of blood samples and tissue for histo-pathological examination

Blood samples were collected from the retro-orbital plexus of veins of all rats (Sorg and Buckner 1964). Samples were left to clot at room temperature then centrifuged at 1500 rpm for 10 min for serum separation. Serum samples were stored at − 20 °C for analysis of intracellular adhesion molecule 1(ICAM-1), leukotriene B4 (LTB4) and interleukinβ 4 (ILβ4) levels, which were performed according to manufacturer’s guidelines.

All groups were humanely sacrificed 3 h after last dose treatment. Skin specimens from each animal were prepared and fixed in 10% neutral formalin solution for 24 h, embedded in paraffin and stained with hematoxin–eosin or toluidine blue stain. Specimens were then evaluated for inflammatory cells, mast cells and degranulation. Mastocyte infiltration was expressed as the number of mastocytes (non-degranulated & ± degranulated) at 400 HPF/group (Drury and Wallington 1980).

##### Statistical analysis

Results of biochemical parameters were expressed as means of levels of intracellular adhesion molecule 1(ICAM-1), leukotriene B4 (LTB4) and interleukinβ 4 (ILβ4) ± standard error (SE). Number of samples in each group was 5 (*N* = 5). One-way analysis of variance (ANOVA) was used to compare means, followed by the Tukey–Kramer multiple comparisons test. *P* value ≤ 0.05 was considered significant. Statistical analyses were done by using “Graph pad prism software, version 8”.

## Results and discussion

### Phytochemical screening

The outcomes of the initial phytochemical analysis of extracts from three plants are displayed in Table 1. The findings reveal the existence of flavonoids, carbohydrates, tannins, triterpenoids and/or steroids, alkaloids, Cardiac glycosides, and saponin in *Acacia nilotica* and, *Glycyrrhiza glabra* EtOH extracts(Chauhan et al. 2018). These outcomes are consistent with those reported by Byakod, (Byakod 2023) for the AN extract and Chauhan et al. for the GG extract, although coumarins were identified in our samples. Volatile oil was identified in the extract of *Boswellia sarca*. These results align with those documented by Hussain et al., who noted the presence of volatile oils (Hussain et al. 2013).

**Table 1:** Phytochemical screening of *three plant extract* and their fractions

Groups	BS		AN EtOH extract	GG EtOH extract
	Volatile oil	Pet. ether		
Volatile oils	** + + + **	** + **	**−**	**−**
Carbohydrate	**−**	** + + + **	** + + + **	** + + + **
Tannins	**−**	**−**	** + + + **	** + + + **
Flavonoids, NaOH	**−**	**−**	** + + **	** + + + **
Flavonoids (Shinoda test)	**−**	** + **	** + + **	** + + + **
Saponin	**−**	**−**	** + + + **	** + + + **
Sterol and/or triterpenes	**−**	** + + + **	** + **	** + **
Coumarins	**−**	** + + **	** + **	** + + + **
Alkaloids	**−**	**−**	**−**	** + **

(+ +), (+) and (−) refer to high, low and absente amount, respectively

### Chemical composition of the volatile and fixed oil of *Boswellia sarca* resin

**Fig. 1:**
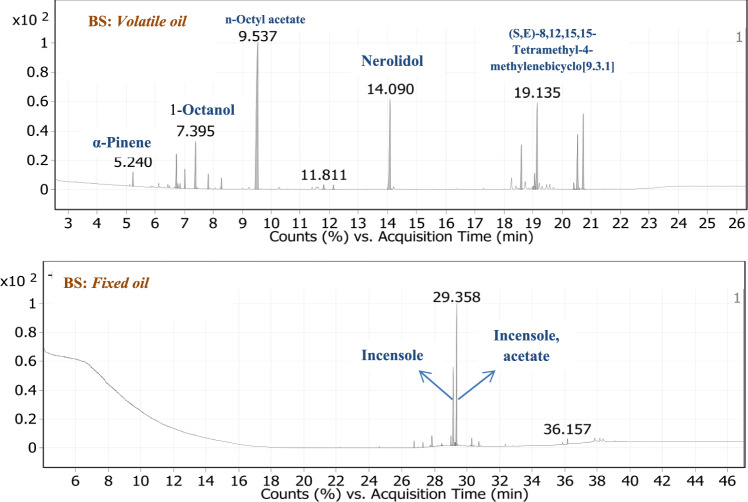
TIC of volatile and fixed oil of BS

The volatile terpenoids and chemical composition of the volatile oil derived from *Boswellia sarca* resin were analyzed using GC/MS techniques. Key compounds identified include 1-Octanol (5.86%), n-Octyl acetate (37.19%), Nerolidol (13.12%), (S, E)−8,12,15,15-Tetramethyl-4-methylenebicyclo[9.3.1]pentadeca-7,11-diene (10.71%), Incensole (7.39%), and Incensole acetate (8.09%) as the primary constituents, as shown in Fig. 1 and Table 2. Additionally, GC/MS analysis of the fixed oil revealed that Incensole acetate (32.44%) and Incensole (50.12%) were the dominant compounds, as detailed in Fig. 1 and Table 2.

**Table 2:** GC/MS of volatile and fixed oil *Boswellia sarca*

Peak	RT	Compounds names	Formula	Area Sum %
BS *Volatile oil extract*				
1	5.24	α-Pinene	C_10_H_16_	1.16
2	6.73	D-Limonene	C_10_H_16_	2.9
3	6.783	Eucalyptol	C_10_H_18_O	0.39
4	6.861	Trans-β-Ocimene	C_10_H_16_	0.41
5	7.027	β-Ocimene	C_10_H_16_	1.56
6	7.395	1-Octanol	C_8_H_18_O	5.86
7	7.828	Linalool	C_10_H_18_O	1.27
8	8.285	2,3,3-trimethyl-1,4-Pentadiene	C_8_H_14_	0.95
9	9.537	**n-Octyl acetate**	**C**_**10**_**H**_**20**_**O**_**2**_	**37.19**
10	11.811	Nerol acetate	C_12_H_20_O_2_	0.57
11	12.137	Decyl acetate	C_12_H_24_O_2_	0.45
12	**14.09**	**Nerolidol**	**C**_**15**_**H**_**26**_**O**	**13.12**
13	18.595	(-)-Cembrene A	C_20_H_32_	5.19
14	18.998	Isoneocembrene A	C_20_H_32_	0.42
15	19.046	Cembrene	C_20_H_32_	1.61
16	**19.135**	**(S,E)−8,12,15,15-Tetramethyl-4-methylenebicyclo[9.3.1]pentadeca-7,11-diene**	**C**_**20**_**H**_**32**_	**10.71**
17	20.393	Nephthenol	C_20_H_34_O	0.76
18	20.53	Incensole	C_20_H_34_O_2_	7.39
19	20.72	**Incensole, acetate**	C_22_H_36_O_3_	8.09
BS *Fixed oil extract*				
1	26.747	Hexadecanoic acid, methyl ester	C_17_H_34_O_2_	1.68
2	27.294	1,5,9-Cyclotetradecatriene	C_20_H_32_	1.42
3	27.84	(S,E)−8,12,15,15-Tetramethyl-4-methylenebicyclo[9.3.1]pentadeca-7,11-diene	C_20_H_32_	3.08
4	28.439	9-Octadecenoic acid, methyl ester	C_19_H_36_O_2_	0.69
5	29.009	(-)-Nephthenol	C_20_H_34_O	3.31
6	29.153	Incensole	C_20_H_34_O_2_	**32.44**
7	29.274	Andrographolide	C_20_H_30_O_5_	1.26
8	29.358	Incensole, acetate	C_22_H_36_O_3_	**50.12**
9	30.283	Incensole oxide, acetate	C_22_H_36_O_4_	2.78
10	30.716	9-(3,3-Dimethyloxiran-2-yl)−2,7-dimethylnona-2,6-dien-1-ol	C_15_H_26_O_2_	1.55
11	36.157	2(1H)Naphthalenone, 3,5,6,7,8,8a-hexahydro-4,8a-dimethyl-6-(1-methylethenyl)-	C_15_H_22_O	1.68

### Phytochemical investigation of *Glycyrrhiza glabra* (GG) ethanol extract

**Fig. 2:**
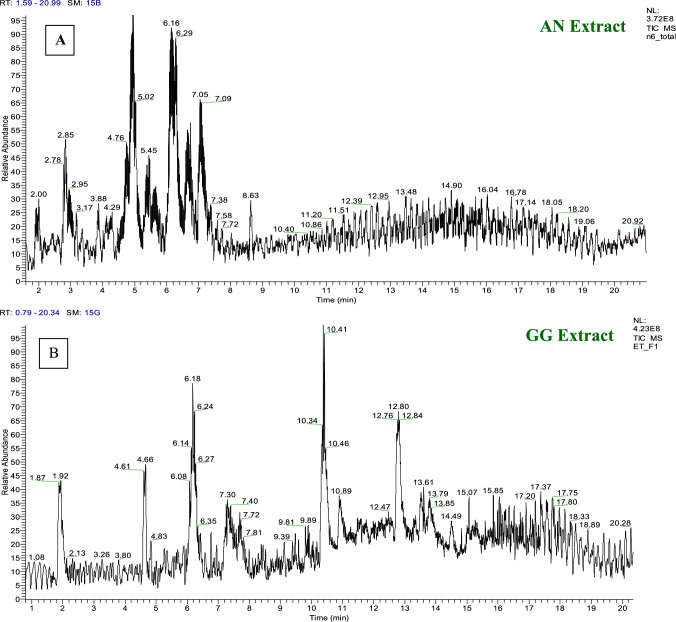
Profiles of listed compounds by LC-MSMS of *AN* and *GG* extract

Phytochemical analysis of the ethanol extract of *Glycyrrhiza glabra* (GG) identified a total of 31 distinct compounds. Among these, key compounds include 2-Acetoxybenzoic acid, Vicenin II, Apigenin 6,8-di-C-glucoside, Scopoletin, 5-Hydroxycoumarin, and Sophoraflavone B, which are known for their antioxidant and anti-inflammatory properties. Other notable compounds such as Isovitexin, Calycosin 7-O-glucoside, and (2S)-Naringenin 6-C-beta-D-glucopyranoside have been linked to various biological activities, including anti-cancer, anti-viral, and cardiovascular benefits. Furthermore, compounds like Isoliquiritigenin 4,4′-diglucoside, Daidzein, and Liquiritinapioside contribute to the therapeutic efficacy of GG by exhibiting immunomodulatory effects. Additional bioactive constituents such as Gancaonin S, Licoriphenone, Glycyrin, and Glyasperin B, along with others, further underscore the ethnomedicinal significance of *Glycyrrhiza glabra* as a source of natural therapeutic agents. These findings highlight the complex chemical diversity of GG and its potential for further pharmacological applications (Fig. 2 and Table 3) .

**Table 3:** Profiles of listed compounds by LC-MSMS of AN and GG plants

No	RT	Compounds names/SMILES	Chemical formula	Mass		∆ ppm	PDA	Ref.
				Measured & calculated	Exact mass of [M-H]-			
AN EtOH extract								
1	2.88	Gallic acid O = C(O)c1cc(O)c(O)c(O)c1	C_7_H_6_O_5_	169.0132, 169.0131	125.0230[C6H5O3]	0.4711	268, 356	a
2		Gallic acid hexoside	C_13_H_16_O_10_	331.0670, 331.0660	271.0461[C11H11O8], 241.0347[C10H9O7], 169.0131[C7H5O5]	3.0599	278, 357	d
3	3.73	Leucocyanidin Oc1cc(O)c2c(c1)OC(c1ccc(O)c(O)c1)C(O)C2O	C_15_H_14_O_7_	305.0672, 305.0656	261.0779[C14H13O5], 219.0779[C12H11O4], 167.0339[C8H7O4], 125.0230[C6H5O3]	5.4120	276, 345	b
4	3.84	Gentisic acid 2-beta-D-glucoside C1 = CC(= C(C = C1O)C(= O)O)OC2 C(C(C(C(O2)CO)O)O)O	C_13_H_16_O_9_	315.0724, 315.0711	165.0175[C8H5O4], 152.0103[C7H4O4], 108.0201[C6H4O2]	4.1220	279, 345	c
5	4.03	(+)-Gallocatechin Oc1cc(O)c2c(c1)O[C@H](c1cc(O)c(O)c(O)c1)[C@@H](O)C2	C_15_H_14_O_7_	305.0664, 305.0656	261.0768[C14H13O5], 219.0657[C12H11O4], 167.0339[C8H7O4], 125.0229[C6H5O3]	2.6110	274, 345	d
6	4.25	(-)-Epigallocatechin Oc1cc(O)c2c(c1)O[C@H](c1cc(O)c(O)c(O)c1)[C@H](O)C2	C_15_H_14_O_7_	305.0671, 305.0656	261.0770[C14H13O5], 219.0656[C12H11O4], 167.0338[C8H7O4], 125.0231[C6H5O3]	5.1119	–	b
7	4.29	Tryptophan NC(Cc1c[nH]c2ccccc12)C(= O)O	C_11_H_12_N_2_O2	203.0820, 203.0815	159.0915[C10H11 N2], 116.0491[C8H6 N]	2.5956	278	a
8	4.67	Hydroxyferulic acid COc1cc(/C = C/C(= O)O)cc(O)c1O	C_10_H_10_O_5_	209.0449, 209.0444	165.0545[C9H9O3], 121.0642[C8H9O]	2.0389	280, 359	a
9	4.85	Catechin Oc1cc(O)c2c(c1)O[C@H](c1ccc(O)c(O)c1)[C@@H](O)C2	C_15_H_14_O_6_	289.0717, 289.0707	245.0818[C14H13O4], 205.0502[C11H9O4], 151.0390[C8H7O3], 109.0280[C6H5O2]	3.8833	279, 374	b
10	5.38	Dihydroquercetin c1(cc(c2c(c1)O[C@@H]([C@H](C2 = O)O)c1cc(c(cc1)O)O)O)O	C_15_H_12_O_7_	303.0486, 303.0499	285.0407[C15H9O6], 165.0181[C8H5O4], 137.0231[C7H5O3], 125.0229[C6H5O3]	− 4.3380	280, 346	b
11	5.74	(-)-Epicatechin Oc1cc(O)c2c(c1)O[C@H](c1ccc(O)c(O)c1)[C@H](O)C2	C_15_H_14_O_6_	289.0718, 289.0707	245.0820[C14H13O4], 151.0388[C8H7O3], 125.0230[C6H5O3]	4.0605	279, 374	b
12	6.19	Isoquercitrin O = c1c(O[C@@H]2O[C@H](CO)[C@@H](O)C(O)C2O)c(-c2ccc(O)c(O) c2)oc2cc(O)cc(O)c12	C_21_H_20_O_12_	463.0887, 463.0871	300.0274[C15H8O7], 271.0251[C14H7O6], 137.0230[C7H5O3]	3.5474	280, 346	b
13	6.83	Genistein O = c1c(-c2ccc(O)cc2)coc2cc(O)cc(O)c12	C_15_H_10_O_5_	269.0458, 269.0444	133.0283[C8H5O2]	5.0438	244, 279	a
14	6.89	Quercetin 3-O-alpha-L-rhamnoside c1(cc(c2c(c1)oc(c(c2 = O)O[C@@H]1O[C@H]([C@@H]([C@H]([C@@H]1O)O)O)C)c1ccc(c(c1)O)O)O)O	C_21_H_20_O_11_	447.0932, 447.0922	313.9644[C14H12O9], 285.0395[C15H9O6]	2.1973	279, 340	b
15	7.26	Phloretin-2′-O-glucoside C1 = CC(= CC = C1 CCC(= O)C2 = C(C = C(C = C2O[C@H]3[C@@H]([C@H]([C@@H]([C@H](O3)CO)O)O)O)O)O)O	C_21_H_24_O_10_	435.1295, 435.1286	341.0665[C18H13O7], 273.0770[C15H13O5], 179.0341[C9H7O4], 167.0339[C8H7O4]	2.8692	249, 279	c
16	7.76	Isoliquiritigenin O = C(/C = C/c1ccc(O)cc1)c1ccc(O)cc1O	C_15_H_12_O_4_	255.0659, 255.0652	153.0182[C7H5O4], 135.0075[C7H3O3], 91.0175[C6H3O]	2.9322	246, 278	d
17	7.93	Kaempferol O = c1c(O)c(-c2ccc(O)cc2)oc2cc(O)cc(O)c12	C_15_H_10_O_6_	285.0404, 285.0394	270.0534[C15H10O5], 150.03089[C8H6O3] 175.0392[C10H7O5]	3.7585	–	b
18	8.11	Delphinidin C1 = C(C = C(C(= C1O)O)O)C2 = [O +]C3 = CC(= CC(= C3 C = C2O)O)O	C_15_H_10_O_7_ +	301.0347, 301.0343	178.9975, 151.0024[C8H7O3]	1.4946	276	c
19	8.39	2-Methoxycinnamic acid COC1 = CC = CC = C1/C = C/C(= O)O	C_10_H_10_O_3_	177.0547, 177.0546	145.0284[C9H5O2], 121.0280[C7H5O2]	0.6360	246, 276	c
20	8.57	Naringenin O = C1 C[C@@H](c2ccc(O)cc2)Oc2cc(O)cc(O)c21	C_15_H_12_O_5_	271.0607, 271.0601	177.0187[C9H5O4] 153.0182[C7H5O4], 135.0437[C8H7O2]	2.0992	248	d
GG EtOH extract								
1	5.08	2-Acetoxybenzoic acid O = C(O)C1 = C(OC(C) = O)C = CC = C1	C_9_H_8_O_4_	179.0341, 179.0339	151.0390[C8H7O3], 136.0438[C8H7O2]	1.0482		a
2	5.19	Vicenin c1(c(c(c2c(c1[C@H]1[C@H]([C@H]([C@@H]([C@@H](O1)CO)O)O)O)oc(cc2 = O)c1ccc(cc1)O)O)[C@H]1[C@@H]([C@H]([C@@H]([C@@H](O1)CO)O)O)O)O	C_27_H_30_O_15_	593.1542, 593.1540	473.1076[C23H21O11], 383.0777[C20H15O8], 353.0671[C19H13O7]	− 0.4046	270, 313	a
3	5.34	Scopoletin	C_10_H_8_O_4_	191.0340, 191.0339	–	0.4232	–	b
4	5.37	5-Hydroxycoumarin c1cc(c2c(c1)oc(= O)cc2)O	C_9_H_6_O_3_	161.0233, 161.0233	133.0283[C8H5O2],	− 0.2213	–	b
5	5.93	Sophoraflavone B c1(ccc2c(c1)oc(cc2 = O)c1ccc(cc1)O[C@H]1[C@@H]([C@H]([C@@H]([C@H](O1)CO)O)O)O)O	C_21_H_20_O_9_	415.1037, 415.1024	253.0504[C15H9O4]	3.2285	284	d
6	6.07	Isovitexin c1(c(c(c2c(c1)oc(cc2 = O)c1ccc(cc1)O)O)[C@H]1[C@@H]([C@H]([C@@H]([C@H](O1)CO)O)O)O)O	C_21_H_20_O_10_	431.0957, 431.0973	341.0670[C18H13O3], 311.0562[C17H11O6], 283.0610[C16H11O5]	− 3.6420	282, 303	b
7	6.69	Calycosin 7-O-glucoside O(c1cc2c(c(= O)c(co2)c2ccc(c(c2)O)OC)cc1)[C@H]1[C@H]([C@H]([C@@H]([C@H](O1)CO)O)O)O	C_22_H_22_O_10_	445.1127, 445.1129	283.0618[C16H11O5], 268.0367[C15H8O5]	− 0.5669	240, 275	b
8	6.96	Hemiphloin c1(c(c(c2c(c1)O[C@@H](CC2 = O)c1ccc(cc1)O)O)[C@H]1[C@@H]([C@H]([C@@H]([C@H](O1)CO)O)O)O)O	C_2_1H_22_O_10_	433.1133, 433.1129	313.0724[C17H13O6], 271.0612[C15H11O5], 151.0024[C7H3O4]	0.8971	278, 311	c
9	7.50	Liquiritinapioside [C@@H]1(Oc2c(C(= O)C1)ccc(c2)O)c1ccc(O[C@@H]2O[C@H]([C@H]([C@@H]([C@H]2O[C@H]2[C@H]([C@@](CO2)(O)CO)O)O)O)CO)cc1	C_26_H_30_O_13_	549.1616, 549.1603	429.1039[C18H21O13], 255.0662[C15H11O4]	2.3541	245, 325	c
10	7.30	Isoliquiritigenin 4,4′-diglucoside c1(cc(c(cc1)C(= O)/C = C/c1ccc(cc1)O[C@@H]1O[C@H]([C@H]([C@H]([C@@H]1O)O)O)CO)O)O[C@H]1[C@@H]([C@H]([C@@H]([C@@H](O1)CO)O)O)O	C_27_H_32_O_14_	579.1782, 579.1779	285.0775[C16H13O5], 355.0666[C15H11O4]	− 0.5180	–	b
11	7.33	Daidzein	C_15_H_10_O_4_	253.0504, 253.0495	253.0609[C12H11O5], 191.0704[C11H11O3], 153.0073[C7H5O4]	3.4767	–	b
12	7.51	Pinocembroside c1(cc2c(c(c1)O)C(= O)C[C@H](O2)c1ccccc1)O[C@H]1[C@@H]([C@H]([C@@H]([C@H](O1)CO)O)O)O	C_21_H_22_O_9_	417.1182, 417.1180	297.0775[C17H13O5], 255.0662[C15H11O4], 153.0182[C7H5O4], 135.0074[C7H3O3]	0.3726	245, 282	d
13	8.78	Gancaonin S c1(c(cc(c(c1O)CC = C(C)C)O)CCc1ccc(c(c1)O)O)CC = C(C)C	C_24_H_3_0O_4_	383.1143, 383.1125	368.0894[C20H16O7], 311.0562[C17H11O6]	4.5116	–	d
14	10.15	Licoriphenone c1(cc(c(cc1)C(= O)Cc1c(c(c(cc1O)OC)CC = C(C)C)OC)O)O	C_21_H_24_O_6_	371.1506, 371.1489	339.0865[C19H15O6], 181.0495[C9H9O4]	4.4695	–	b
15	11.16	Kanzonol T 5,7,2′-Trihydroxy-6-(3-hydroxy-3-methylbutyl)−6″,6″-dimethylpyrano[2″,3″:4′,3′]isoflavone c1(c(c(c2c(c1)occ(c2 = O)c1c(c2c(cc1)OC(C = C2)(C)C)O)O)CCC(O)(C)C)O	C_25_H_26_O_7_	437.1606, 437.1595	201.0915[C13H13O2]	2.6649	250, 280	d
16	12.38	Glycyrin 3-(2,4-Dihydroxyphenyl)−5,7-dimethoxy-6-prenylcoumarin	C_22_H_22_O_6_	381.1340, 381.1333	363.0873[C21H15O6], 323.0544[C18H11O6], 161.0230[C9H5O3]	1.8559	256, 280	d
17	12.63	Scanderone 4′,5-Dihydroxy-3′-prenyl-2″,2″-dimethylchromeno[7,8:6″,5″]isoflavone c1c2c(c3c(c1O)c(= O)c(co3)c1ccc(c(c1)CC = C(C)C)O)C = CC(O2)(C)C	C_25_H_24_O_5_	403.1541, 403.1540	335.0925[C20H15O5], 201.0910[C13H13O2], 135.0439[C8H7O2]	0.2058	245, 282	d
18	12.83	Glyinflanin A c1(c(cc(c(c1)O)C(= O)/C = C(/c1cc(c(cc1)O)CC = C(C)C)\O)CC = C(C)C)O	C_25_H_28_O_5_	407.1859, 407.1853	379.1916[C24H27O4], 310.1205[C19H18O4], 203.0706[C12H11O3], 177.0911[C11H13O2]	1.3548	280, 311	
19	12.99	Kanzonol Y 4,2′,4′,alpha-Tetrahydroxy-3,5′-diprenyldihydrochalcone c1(c(cc(c(c1)C(= O)[C@@H](Cc1cc(c(cc1)O)CC = C(C)C)O)O)O)CC = C(C)C	C_25_H_30_O_5_	409.2019, 409.2010	235.0972[C13H15O4], 217.0863[C13H13O3], 177.0910[C11H13O2], 135.0437[C8H7O2]	2.2564	268, 280	d
20	13.54	Glyasperin B 5,2′,4′-Trihydroxy-7-methoxy-6-prenylisoflavanone c1(c(c(c2c(c1)OC[C@@H](C2 = O)c1c(cc(cc1)O)O)O)CC = C(C)C)OC	C_21_H_22_O_6_	369.1315, 369.1333	311.0919[C18H15O5], 247.0974[C14H15O4], 207.1018[C12H15O3], 161.0234[C9H5O3]	− 4.8630	–	d
21	13.80	Hirtellanine I OC1 = CC2 = C(C = C1)C(C(C3 = C(OC)C(C = CC(C)(C)O4) = C4 C = C3O) = CO2) = O	C_21_H_18_O_6_	365.1032, 365.1020	347.0932[C21H15O5], 321.1127[C20H17O4], 165.0183[C8H5O4]	3.3284	–	d
22	13.91	7-O-Methylluteone 5,2′,4′-Trihydroxy-7-methoxy-6-prenylisoflavone c1(c(c(c2c(c1)occ(c2 = O)c1ccc(cc1O)O)O)CC = C(C)C)OC	C_21_H_20_O_6_	367.1176, 367.1176	321.0768[C19H13O5], 163.0390[C9H7O3]	0.0831	256, 280	d
23	13.95	Gancaonin H 5,7,3′-Trihydroxy-6-prenyl-6″,6″-dimethylpyrano[2″,3″:4′,5′]isoflavone c1(c(c(c2c(c1)occ(c2 = O)c1cc(c2c(c1)C = CC(O2)(C)C)O)O)CC = C(C)C)O	C_25_H_24_O_6_	419.1474, 419.1489	351.1222[C21H19O5], 231.1022[C14H15O3], 161.0959[C11H13O]	− 3.5416	256, 279	d
24	14.13	Xambioona c1cc2c(c3c1OC(C = C3)(C)C)O[C@H](CC2 = O)c1ccc2c(c1)C = CC(O2)(C)C	C_25_H_24_O_4_	387.1597, 387.1591	161.0443[C6H9O5]	1.6616	254	a
25	14.16	Kanzonol V (5,4′-Dihydroxy-6-prenyl-6″,6″-dimethylpyrano[2″,3″:2′,3′]−2-arylbenzofuran) c1(c(cc2c(c1)cc(o2)c1c2c(c(cc1)O)C = CC(O2)(C)C)O)CC = C(C)C	C_24_H_24_O_4_	375.1602, 375.1591	306.0901[C19H14O4]	3.0163	267, 311	d
26	14.30	Hispaglabridin B c12ccc3c(c1 C = CC(O2)(C)C)OC[C@@H](C3)c1c(c2c(cc1)OC(C = C2)(C)C)O	C_25_H_26_O_4_	389.1764, 389.1747	333.1142[C21H17O4], 119.0335[C4H7O4]	4.3332	278	d
27	14.69	Glabraisoflavanone A CC(O1)(C)CCC2 = C1 C = CC([C@@]3([H])C(C(C = CC(O) = C4 C/C = C(C)/C) = C4OC3) = O) = C2	C_25_H_28_O_4_	391.1914, 391.1904	203.1071[C13H15O2]	2.5305	279	d
28	15.86	Liquoric acid	C_30_H_44_O_5_	483.3121, 483.3105	439.3218[C29H43O3]	3.2523	279	d
29	17.07	Glabranin c1(cc(c2c(c1 CC = C(C)C)O[C@@H](CC2 = O)c1ccccc1)O)O	C_20_H_20_O_4_	323.1284, 323.1278	201.0920[C13H13O2], 135.0436[C8H7O2]	2.0516	–	d
30	17.14	Gancaonin U (1,3-Diisopentenyl-2,4,6,7-tetrahydroxy-9,10-dihydrophenanthrene) c1(c(cc2c(c1)c1c(CC2)c(c(c(c1O)CC = C(C)C)O)CC = C(C)C)O)O	C_24_H_28_O_4_	379.1904, 379.1904	311.1689	0.0352	–	d
31	17.49	Anaphalisoleanenoic acid O = C(O)[C@@]1(C)CC[C@]2(C)CC[C@@]3(C)[C@]4(C)CC[C@@]5([H])C(C)(C)[C@@H](O)CC[C@]5(C)[C@@]4([H])CC = C3[C@]2([H])C1	C_30_H_48_O_3_	455.3522, 455.3520	–	0.5085	270	d

*The letter “a” refers to identification using the MS-Dial library, “b” indicates identification using the Knapsack library, and “c” refers to using the PubChem database and “d” denotes tentative identification

### Phytochemical profile of *Acacia nilotica* extracts

*Acacia nilotica* extracts are rich in a diverse range of 20 primary metabolites, which play essential roles in cell biosynthetic pathways. These primary metabolites were identified using LC–MS, and their mass spectra were compared to databases such as MS-Dial and the Knapsack library, as well as tentative identification methods. In addition to these primary metabolites, a comprehensive analysis revealed a variety of secondary metabolites, including polyphenolic acids, vitamins, amino acids, and other bioactive compounds. Key identified compounds include Gallic acid, Gallic acid hexoside, Leucocyanidin, (+)−2,3-trans-3,4-cis-3,4,5,7,3’,4’-hexahydroxyflavan, Gentisic acid 2-beta-D-glucoside, and (+)-Gallocatechin. Other secondary metabolites such as (−)-Epigallocatechin, Tryptophan, Hydroxyferulic acid, Catechin, Dihydroquercetin, Taxifolin, and Isoquercitrin also contribute to the chemical richness of *Acacia nilotica*. These secondary metabolites are known for their antioxidant, anti-inflammatory, and protective effects, with particular relevance to their potential role in managing conditions such as atopic dermatitis. The chemical profile of *Acacia nilotica* shares compositional similarities with the findings of Maldini et al., (Maldini et al. 2011), further validating its therapeutic potential (Fig. 2 and Table 3).

### In vivo pharmacological study

#### Grading of Allergic reactions

**Fig. 3:**
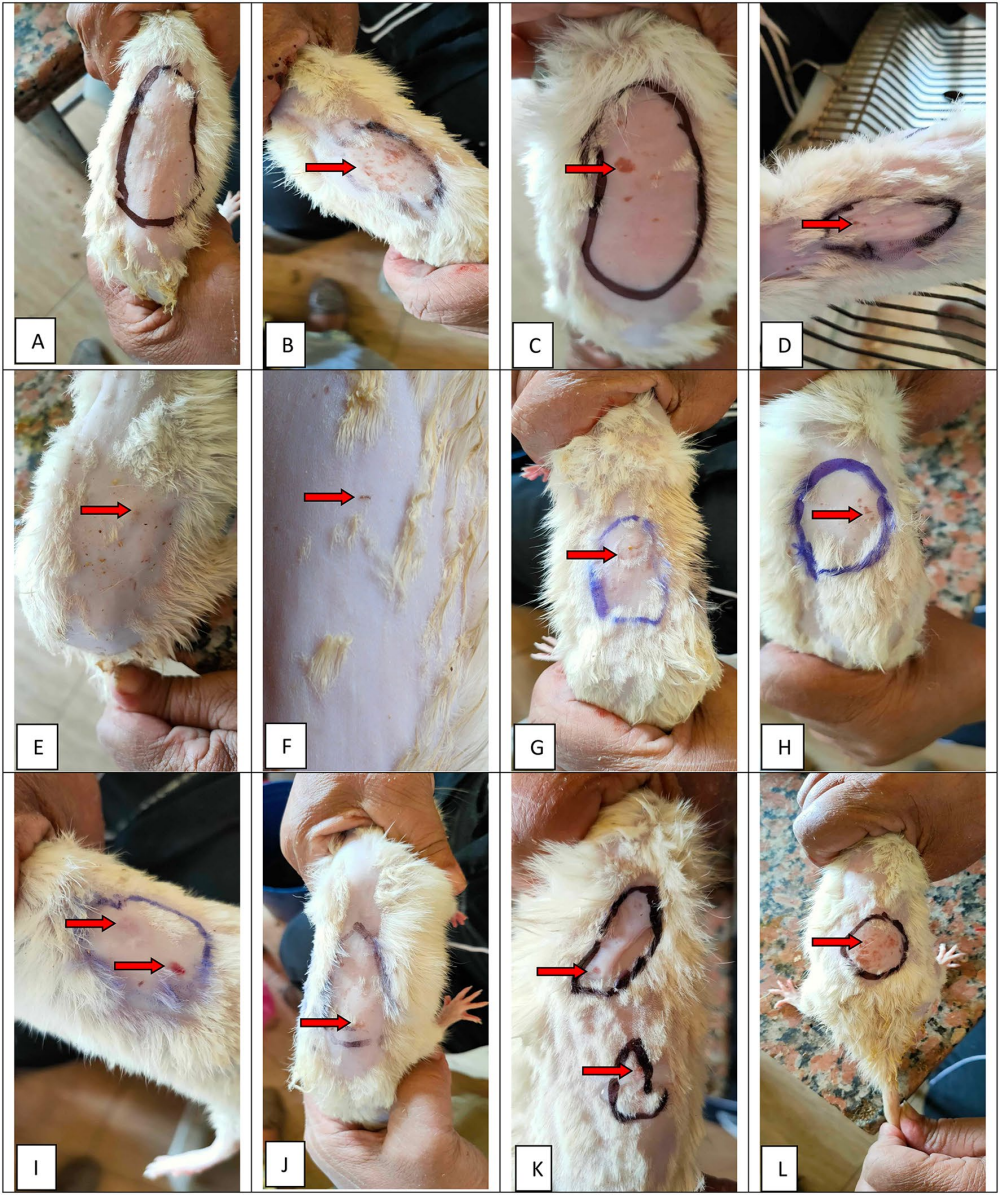
Evaluation of allergic reaction: **A** Normal control, **B**, **C** Positive control, **D** Betamethasone, **E** BS volatile oil, **F**, **G** BS fixed oil, **H**, **I** GG extract, **J**–**L** AN extract. Red arrows point to sites of inflammation in the form of redness, oedema, itching and injuries

In the present study, inspection of skins of 100% of the normal control rats did not show any changes throughout the 180 min duration of the experiment, and were graded as none (Fig. 3a).

On the other hand, the onset of signs of allergy appeared in all histamine-injected groups after 30 min, but their intensity varied after the application of treatment which was done 1 h after injection of histamine, the grading was as follows: 60% of the positive control group that did not receive treatment, were graded as severe and exhibited deep scratches with surrounding redness, while only 40% were graded as moderate (Fig. 3b, c). 80% of rats in the groups that were treated with betamethasone, *Boswellia* volatile and fixed oils were mild, while 20% were graded as moderate and showed deep redness or superficial scratches or both in all three groups (Fig. 3d–g). 60%, *Glycyrrhiza glabra* were graded as mild and showed faint redness, while 40% were graded as moderate (Fig. 3h–i). 40% of the rats in the *Acacia nilotica* group were graded as mild, while 40% were moderate and 20% were severe (Fig. 3j–l).

#### Results of allergic and inflammatory biomarkers

**Fig. 4:**
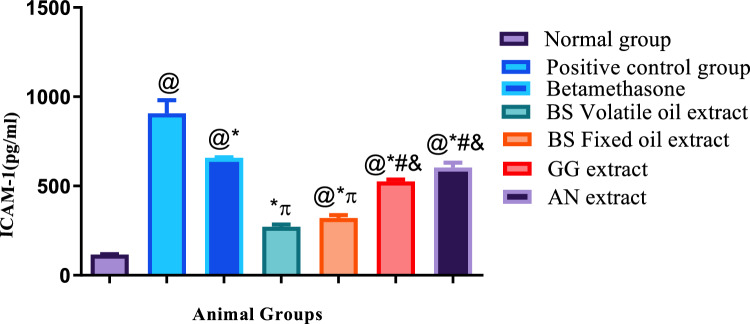
Results of ICAM-1 + SE, *N* = 5, ANOVA test was used to compare means, followed by the Tukey–Kramer multiple comparisons test. *P* ≤ 0.05. @Significant difference from negative control group,*Significant difference from positive control group,  Significant difference from Betamethasone group, #Significant difference from Volatile oil of Frakisence group,&Significant difference from Fixed oil of Frakisence group

**Fig. 5:**
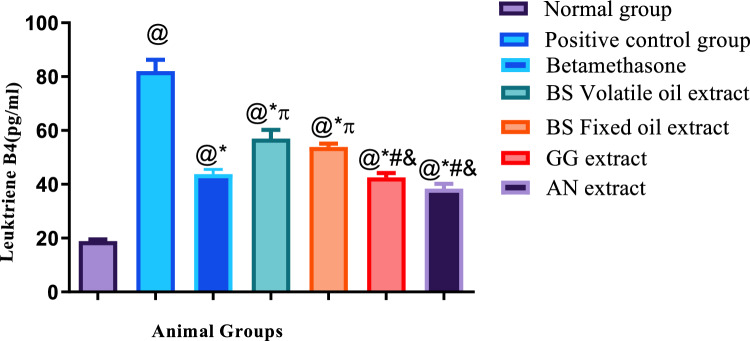
Results of Leukotriene B4 + SE, *N* = 5, ANOVA test was used to compare means, followed by the Tukey–Kramer multiple comparisons test. *P* ≤ 0.05. @Significant difference from negative control group,* Significant difference from positive control group,  Significant difference from Betamethasone group, #Significant difference from Volatile oil of Frakisence group,&Significant difference from Fixed oil of Frakisence group

**Fig. 6:**
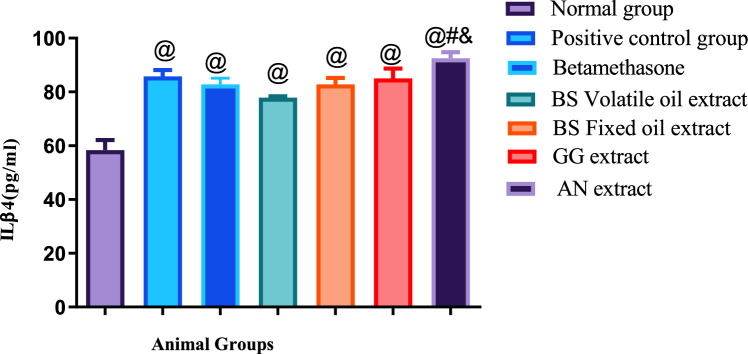
Results of IL 4 + SE, *N* = 5, ANOVA test was used to compare means, followed by the Tukey–Kramer multiple comparisons test. *P* ≤ 0.05. @Significant difference from negative control group,#Significant difference from Volatile oil of Frakisence group,&Significant difference from Fixed oil of Frakisence group

Illustrated in Figs. 4, 5, 6, showed that histamine subcutaneous injection significantly elevated the allergic biomarv0 + kers ICAM −1and Leukotriene B4, as well as the inflammatory biomarker interleukin β4 in all groups, when compared to the normal control group.

Treatment with the standard medication betamethasone cream, as well as volatile and fixed oils of *Boswellia*, extract of *Glycyrrhiza glabra*, and extract of *Acacia nilotica* significantly reduced the allergic biomarkers ICAM-1 and leukotriene B4 in all groups when compared to the untreated positive control group. Yet, the results of the measured biochemical parameters of the treated groups, were significantly higher than the negative control group, except for the ICAM-1 level of the group treated volatile oil of *Boswellia,* as treatment with this natural product exhibited the lowest level of ICAM-1 which was insignificantly different from the negative control group.

Regarding the level of ICAM-1, the effect of treatment with volatile and fixed oils of *Boswellia* was better than the standard medication betamethasone, and showed significant lower levels of ICAM-1, on the other hand, the levels of leukotriene B4 were significantly higher in groups treated with both oils, when compared to the standard medication.

The groups treated with *Glycyrrhiza glabra* and *Acacia nilotica* extracts showed significantly higher levels of ICAM-1 when compared to the groups that were treated with volatile and fixed oils of *Boswellia,* yet the same groups showed significant lower levels of leukotriene B4, when compared to the groups that were treated with volatile and fixed oils of *Boswellia.*

All treated groups showed significant higher levels of Ilβ4, when compared to the negative control group, and non-significant difference from the positive control group. Additionally, the level of Ilβ4 was significantly higher in the group treated with *Acacia nilotica* extract than the groups treated with both *Boswellia* oils.

#### Histopathologic and immuno-histochemical results

The results of the biochemical parameters were consistent with the results of the histopathologic examination where specimens were stained with hematoxylin and eosin (H&E), the results were illustrated in Figs. 7, 8, 9, 10, 11, 12, 13, and were confirmed with immune-histochemical staining with toluidine illustrated in Figs. 14, 15, 16, 17, 18, 19, 20.

**Fig. 7:**
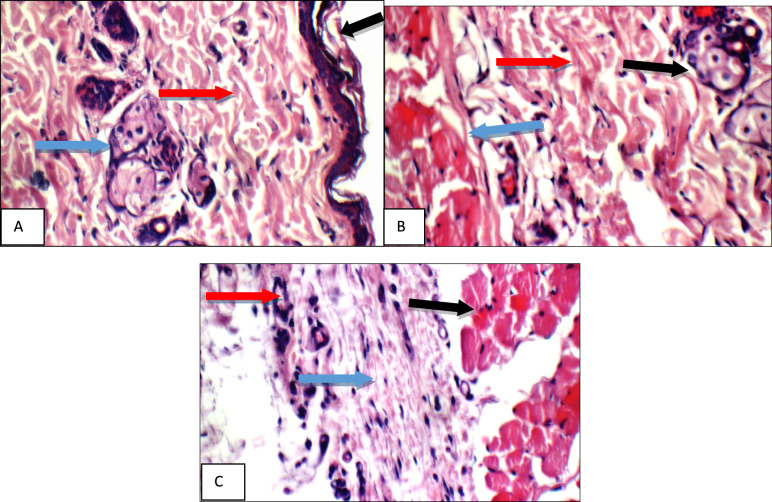
Normal control group: High power view showing **a** average keratinized epidermis (black arrow), average pilo-sebaceous units (blue arrows), and average collagen distribution (red arrow), **b** average pilo-sebaceous units (black arrow), average collagen distribution (red arrow), and average muscles (blue arrows), **c** average muscles (black arrow), and average subcutis (blue arrow) with average blood vessels (red arrow) (H&E X 400)

**Fig. 8:**
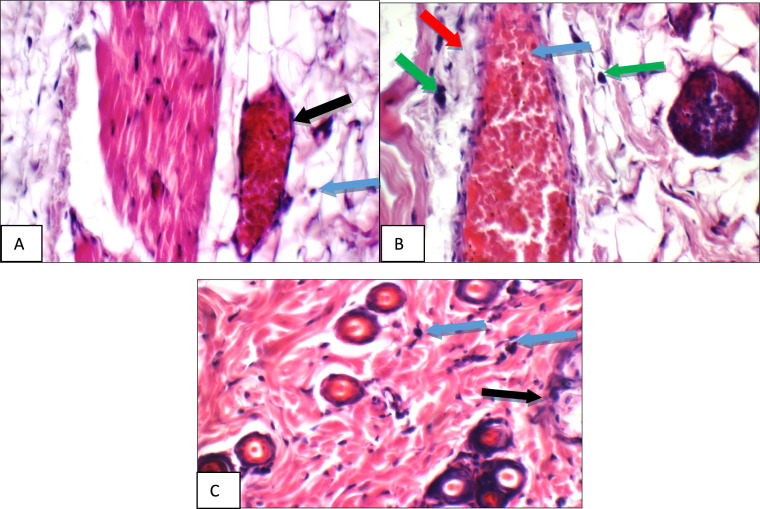
Positive control group: High power view showing **a** markedly dilated congested deep blood vessel (black arrow) with deep inflammatory infiltrate composed mainly of mast cells (blue arrow), **b** markedly dilated congested deep blood vessel (blue arrow) with mild peri-vascular edema (red arrow) and inflammatory infiltrate composed mainly of mast cells (green arrow), **c** moderate peri-adnexal inflammatory infiltrate (black arrow) composed mainly of mast cells (blue arrow) (H&E X 400)

**Fig. 9:**
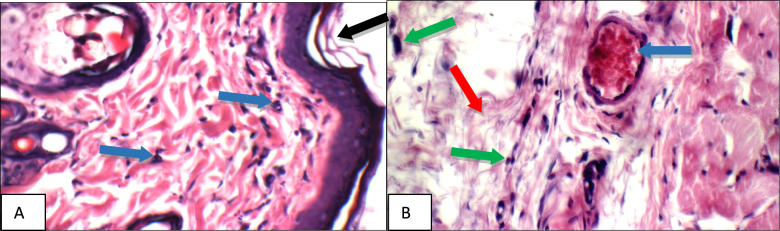
Betamethasone group: High power view showing **a** average keratinized epidermis (black arrow), with moderate superficial inflammatory infiltrate composed mainly of mast cells (blue arrows), **b** mildly dilated congested deep blood vessel (blue arrow), mild edema (red arrow), and mild deepinflammatory infiltrate composed mainly of mast cells (green arrows) (H&E X 400)

**Fig. 10:**
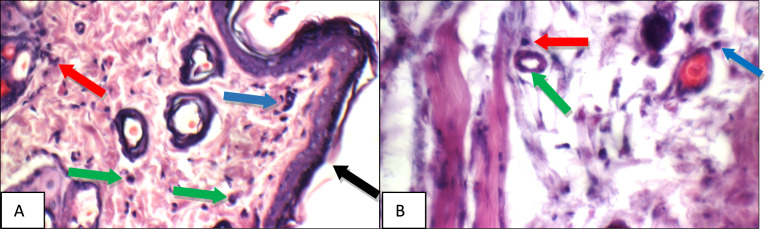
BS volatile oil: High power view showing **a** intact keratinized epidermis (black arrow), with mild superficial (blue arrows) and peri-adnexal (red arrow) inflammatory infiltrate composed mainly of mast cells (green arrow), **b** mild peri-adnexal(blue) and deep inflammatory infiltrate composed mainly of mast cells (red arrow), and average deep blood vessel (green arrow) (H&E X 400)

**Fig. 11:**
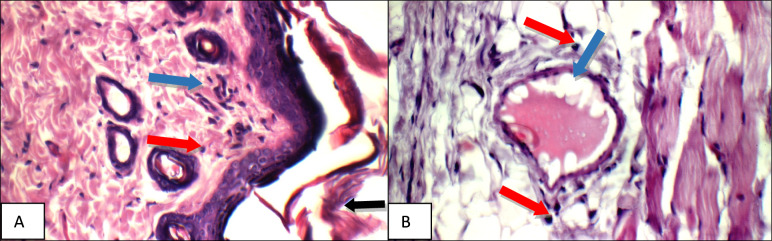
BS fixed oil: High power view showing **a** intact keratinized epidermis (black arrow), with mild superficial (blue arrows) and peri-adnexal inflammatory infiltrate (red arrow), **b** mildly dilated congested deep blood vessel (blue arrow) with peri-vascular inflammatory infiltrate composed mainly of mast cells (red arrow) (H&E X 400)

**Fig. 12:**
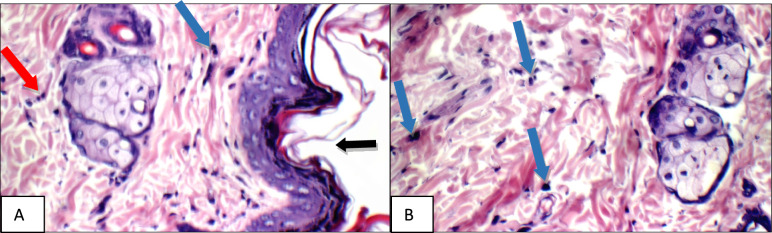
GG extract: High power view showing **a** intact keratinized epidermis (black arrow), mild superficial (blue arrows) and peri-adnexal inflammatory infiltrate (red arrow), **b** mild deep inflammatory infiltrate composed mainly of mast cells (blue arrows) (H&E X 400)

**Fig. 13:**
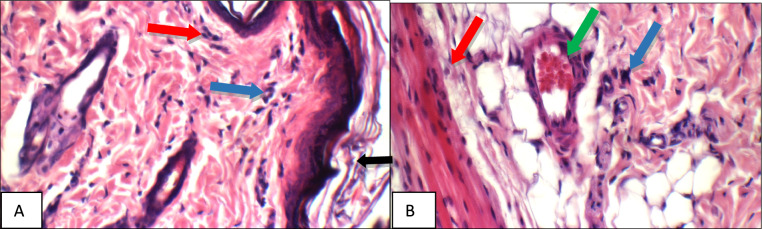
AN extract: High power view showing **a** intact keratinized epidermis (black arrow), moderate superficial (blue arrow) and peri-adnexal inflammatory infiltrate (red arrow), **b** moderate peri-adnexal(blue arrow) and deep intra-muscular inflammatory infiltrate (red arrow) with mildly dilated congested blood vessel (green arrow) (H&E X 400)

**Fig. 14:**
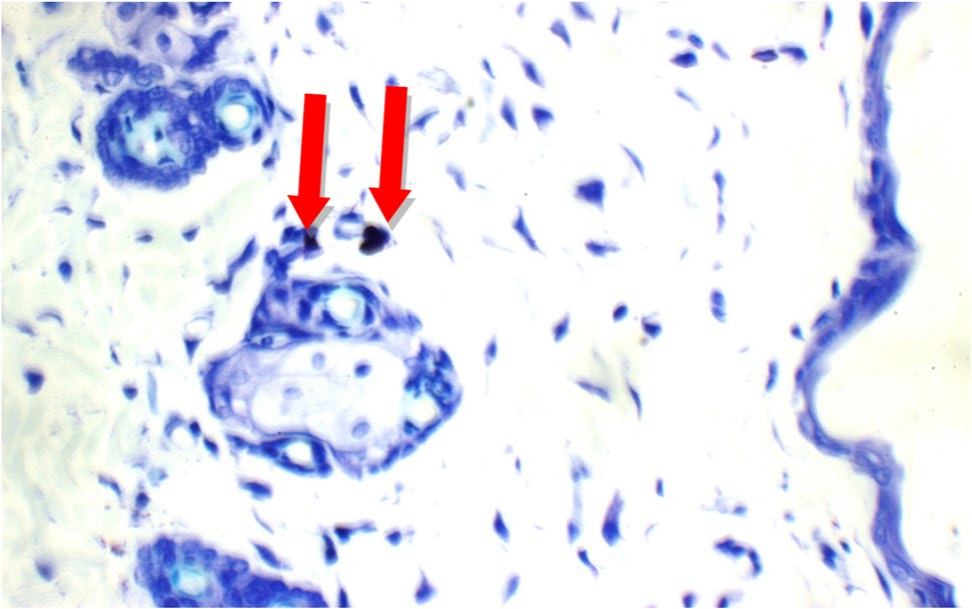
Normal Control: Skin showing o mast cells in superficial dermis, and 2 mast cells/HPF in peri-adnexal area (red arrows) (Toluidine blue stain X 400)

**Fig. 15:**
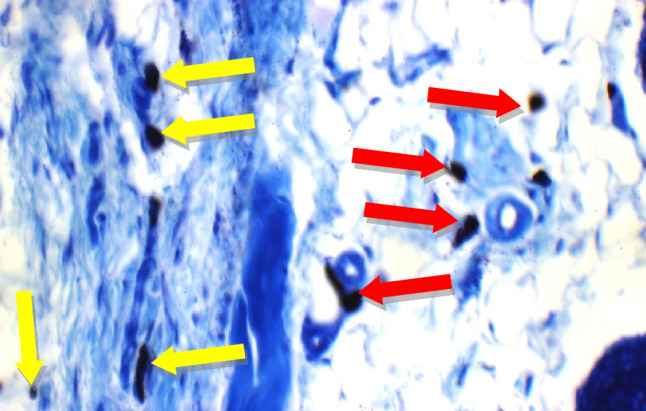
Positive Control: Skin showing 7 mast cells/HPF in peri-adnexal area (red arrows) and 6 mast cells/HPF in deep subcutis are (yellow arrows) (Toluidine blue stain X 400)

**Fig. 16:**
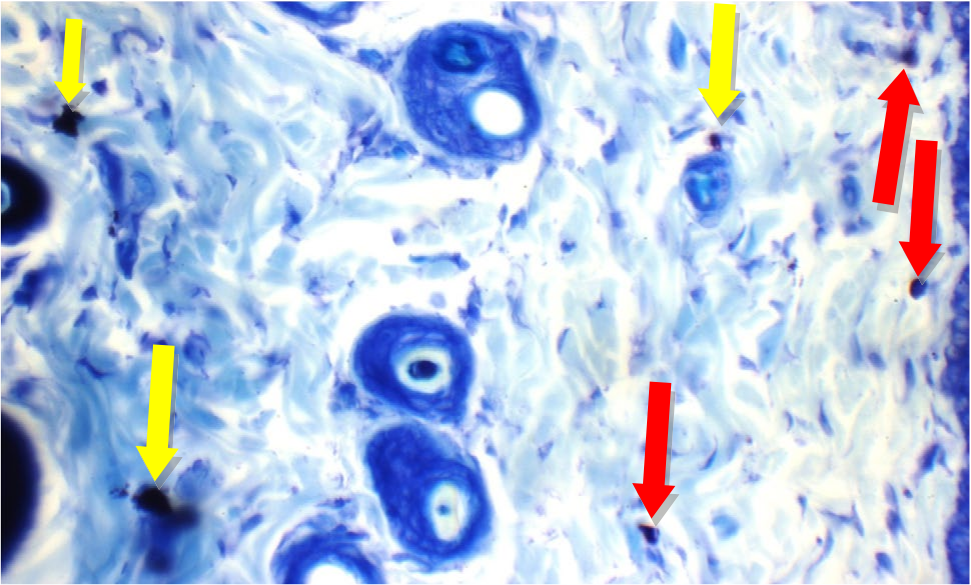
Betamethasone group: Skin showing 3 mast cells/HPF in superficial dermis (red arrow) and 3 in peri-adnexal area (yellow arrows) (Toluidine blue stain X 400)

**Fig. 17:**
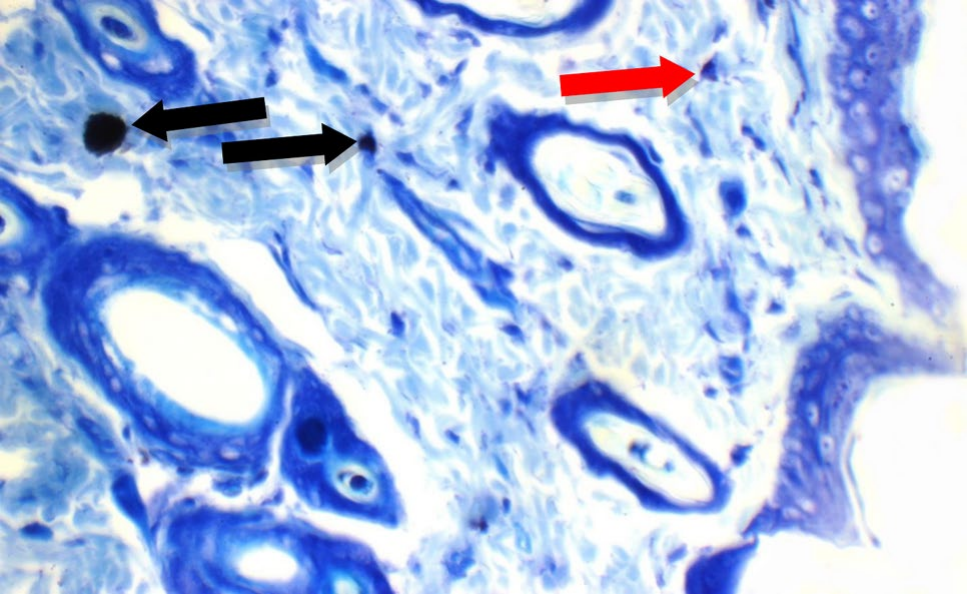
BS volatile oil: Skin showing 1 mast cells/HPF in superficial dermis (red arrow) and 2 in peri-adnexal area (black arrow) (Toluidine blue stain X 400)

**Fig. 18:**
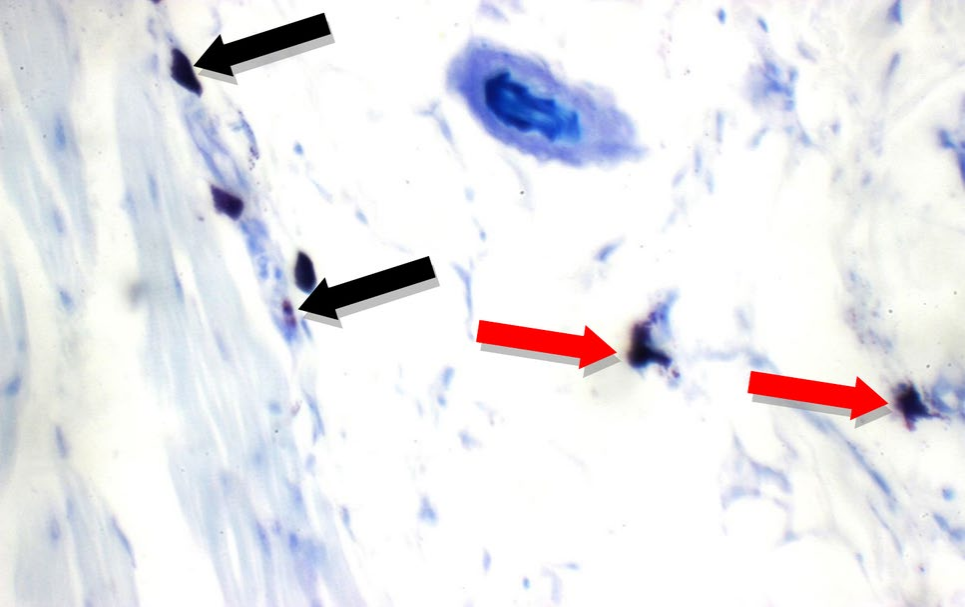
BS fixed oil: Skin showing 2 mast cells/HPF in peri-adnexal area (red arrow) and 4 mast cells/HPF in deep subcutis area (black arrow) (Toluidine blue stain X 400)

**Fig. 19:**
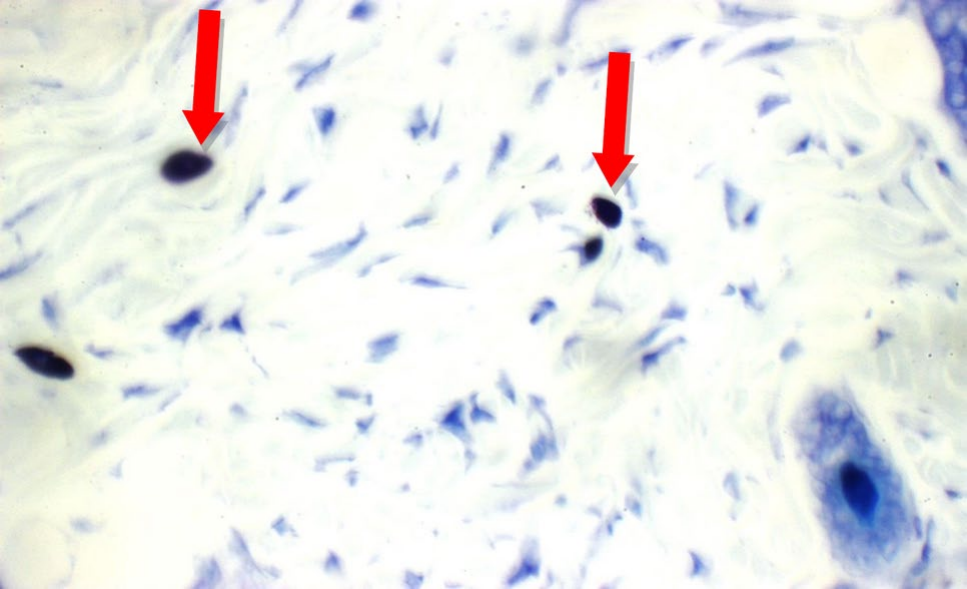
GG extract: Skin showing no mast cells in superficial dermis, and 4 mast cells/HPF in peri-adnexal area (red arrows) (Toluidine blue stain X 400)

**Fig. 20:**
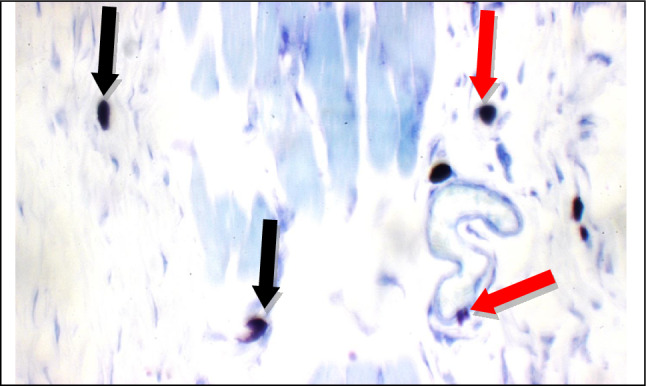
AN extract: Skin showing 5 mast cells/HPF in peri-adnexal area (red arrows), and 2 mast cells/HPF in deep subcutis area (black arrows) (Toluidine blue stain X 400)

High power examination of normal group skin specimens stained with H & E, showed average keratinized epidermis, pilo-sebaceous units, collagen distribution, pilo-sebaceous units, collagen distribution, muscles, average muscles, average subcutis and average blood vessels (Fig. 7a, b, c). On the other hand, high power examination of the positive control group skin specimens stained with H & E, showed markedly dilated congested deep blood vessel with deep inflammatory infiltrate composed mainly of mast cells, mild peri-vascular edema, and moderate peri-adnexal inflammatory infiltrate composed mainly of mast cells (Fig. 8a, b, c).

While, high power examination of betamethasone group skin specimens stained with H & E, showed average keratinized epidermis with moderate superficial and mild deep inflammatory infiltrates composed mainly of mast cells, mildly dilated congested deep blood vessel and mild edema (Fig. 9a–b).

High power examination of *Boswellia* volatile oil group skin specimens stained with H & E, showed intact keratinized epidermis with mild superficial and peri-adnexal inflammatory infiltrate composed mainly of mast cells, and average deep blood vessel (Fig. 10a–b). Also high power examination of *Boswellia* fixed oil group skin specimens stained with H & E, showed intact keratinized epidermis, mild superficial, peri-adnexal and deep inflammatory infiltrates composed mainly of mast cells, but with markedly dilated congested deep blood vessels with mild perivascular edema (Fig. 11a–b).

High power examination of, *Glycyrrhiza glabra* group skin specimens stained with H & E, showed intact keratinized epidermis, mild superficial and peri-adnexal inflammatory infiltrate, and mild deep inflammatory infiltrate composed mainly of mast cells (Fig. 12a–b).

High power examination of *Acacia* group skin specimens stained with H & E, showed intact keratinized epidermis, moderate superficial and peri-adnexal inflammatory infiltrate, deep intra-muscular inflammatory infiltrate with mildly dilated congested blood vessel (Fig. 13a–b).

Additional immuno-histochemical examination with toluidine blue stain of skin specimens in all group, showed no mast cells in and 2 mast cells/HPF in peri-adnexal area in the normal control group (Fig. 14), on the other hand, the positive control group skin specimens showed 7 mast cells/HPF in peri-adnexal area and 6 mast cells/HPF in deep subcutis area (Fig. 15). While the group treated with betamethasone showed 3 mast cells/HPF in superficial dermis and 3 in peri-adnexal area (Fig. 16), that was treated with *Boswellia sarca* volatile oil showed 1 mast cells/HPF in superficial dermis and 2 in peri-adnexal area (Fig. 17), and that was treated with *Boswellia* fixed oil showed 2 mast cells/HPF in peri-adnexal area and 4 mast cells/HPF in deep subcutis area (Fig. 18). The group treated with, *Glycyrrhiza glabra* extract showed no mast cells in superficial dermis, and 4 mast cells/HPF in peri-adnexal area (Fig. 19), and the group treated with *Acacia nilotica* extract showed 5 mast cells/HPF in peri-adnexal area, and 2 mast cells/HPF in deep subcutis area (Fig. 20).

There are two hypotheses for the incidence of allergic dermatitis, which are as follows: the first hypothesis is that the inflammatory and allergy-triggers cause dys-functioning and weakness of the skin barrier, a process that enhances the introduction and presentation of allergens and high penetration of microorganisms causing the incidence of allergic and inflammatory reactions. The other hypothesis is that compromised skin barrier is followed by immune dysregulation so allergic dermatitis occurs easily (Leung et al. 2020).

In the current study, the results of gross visual inspection and scoring of allergic reaction were consistent with the results of the biochemical assay of allergic and inflammatory markers as well as the results of histopathologic and immune-histochemical examination.

In the present study, histamine subcutaneous injection in rats, induced allergic dermatitis in the form of itching, scratches, elevated allergic biomarkers ICAM-1 and leukotriene B4, elevated inflammatory biomarker Ilβ4, as well as histopathologic and immune-histochemical changes in the form markedly dilated congested deep blood vessel with deep inflammatory infiltrate composed mainly of mast cells with peri-vascular edema and inflammatory infiltrate composed mainly of mast cells. The effects of histamine in this study, are explained by the fact that activation of mast cells is mediated by histamine, that leads to an inflammatory cascade, which enhances the pathogenesis of IgE-mediated allergic reaction. Moreover, histamine enhances the secretion IL-4, that plays an important role in the immunological pathways causing allergic dermatitis incidence and occurrence of itching (Kowalska and Narbutt 2024).

The majority of allergic dermatitis cases are children with extrinsic phenotype, that occurs as result of sensitization with high IgE levels and dysfunction of skin barrier (Wollenberg et al. 2021). Patients usually suffer from erythema, mainly in the face with rash and severe itching (Hrubisko et al. 2021).

Since management of dermatitis is achieved by the use of intermediate to high doses of topical corticosteroids for a short duration (Tramontana et al. 2023), that’s why in the current study, we used betamethasone that caused significant amelioration of the signs of allergy that induced by histamine injection in rats, within 120 min.

*Boswellia* extracts, owing to their contents of boswellic acid (Iram et al. 2017), stabilize the lysosomal membranes, which is a vital process in controlling inflammation through inhibition of releasing lysosomal constituents of activated neutrophils, it also protects against protein denaturation that occurs in inflammation (Obiștioiu et al. 2023).

*Boswellia* volatile and fixed oils, in the present study, significantly reduced the allergic activity of histamine, this is consistent with the results of Tsai et al. (Tsai et al. 2022), who stated in their study that α-boswellic acid improved erythema, abrasion, and skin desquamation, it also repair the dysfunctional skin barrier, additionally, it reduced mast cell infiltration, decreased MAP kinase expression, and blocked the NF-κB pathway thus reduced inflammation and epidermal thickening and redness, in dermatitis mouse model.

The results of the current study proved that licorice had anti-allergic activity and anti-inflammatory activity as it reduced both LTB4 and ICAM-1 significantly compared to the untreated positive control group. This effect is due to its content of glycyrrhizin and glycyrrhetinic acid. Also, dipotassium glycyrrhizinate which is a salt of glycyrrhetinic acid, was used as a skin conditioning agent that exhibited anti-allergic and anti-inflammatory activities, via inhibition of leukotriene (Leite et al. 2022).

*Acacia nilotica* possesses anti-oxidant and anti-inflammatory effects as it reduced the levels of cytokines in the study of Khalaf et al., (Khalaf et al. 2023) However, in the current study it exhibited the least curative effect among all tested agents, as grading of allergic reactions were the worst among all treatments, together with level of ICAM-1 as well as histopathologic and immune-histochemical profiles. This effect is most probably due to its poor skin barrier penetrating power ICAM-1 is a marker of vascular inflammation that is inducible by exposure to inflammatory mediators (Witkowska 2005). It is highly expressed in dermatitis lesions and vascular endothelial cells of dermatitis patients (Wolkerstorfer et al. 2003). It is very important in the process of migration of leukocytes from blood vessels to tissues and increases due to pro-inflammatory cytokines (Marinović Kulišić et al. 2023).

Leukotriene B4 (LTB4), which is a lipid mediator possesses a potent chemoattractant properties and is highly produced from activated innate immune cells like neutrophils, macrophages, and mast cells. High level of LTB_4_ is detected in allergic dermatitis as it plays an important role in its pathogenesis (Ohnishi et al. 2008). It plays a very important role in cases of acute inflammation as it attracts lymphocytes, it attracts macrophages as well as neutrophils, and promotes adhesion of leukocytes to vascular endothelium (Abeles et al. 2015).

There is a correlation in the protective mechanism against inflammatory diseases that are induced due to initiated expression of LTB4 or high ICAM-1 levels, as it was reported by Aiello et al. (Aiello et al. 2002), that protection against atherosclerosis was achieved by antagonizing LTB4 via modulation of the interaction of CD11b with ICAM-1(Aiello et al. 2002). This correlation was clear in the present study, as all tested topical agents significantly reduced the levels of both ICAM-1 and LTB4 in rat’s serum when compared to the untreated positive control group. This explains the healing of scratches that resulted from itching due to histamine injection, as reduced levels of ICAM-1 and LTB4 reduced itching and inflammation.

Interleukin β−4 expression increases in extrinsic dermatitis (Tokura 2010). Medications that inhibit IL-4 expression can be used for treatment of atopic dermatitis (Gärtner et al. 2023). Unlike their effects on ICAM-1 and leukotriene B4 in the current study, the used topical agents did not affect its level significantly compared to the positive control group. This weak anti-interleukin β−4 effect may be because topically applied agents have insufficient capability of reaching the systemic circulation and poor systemic penetration which isn’t enough to inhibit interleukin β−4 expression.

## Conclusion

Since most of the assessment parameters in the current experimental work, were in harmony with each other, it can be deduced that *Boswellia sarca* oils as well as, *Glycyrrhiza glabra* extract can provide promising soothing and curative agents against allergic dermatitis, that will achieve better patient’s compliance, when compared to the conventional therapy with corticosteroids, putting in mind that they are natural products that are cheaper, more available and have less side effects. Also, the effect of *Acacia nilotica* cannot be denied, yet further studies regarding the route of administration, suitable dose and modification of the delivery system are required to improve its activity. 

## Data Availability

Data are available on reasonable request.

## Abbreviations

AIF: All-ion fragmentation
AN: Acacia nilotica
BS: Boswellia sarca
EIPICO: Egyptian international pharmaceutical industries company
ESI: Electrospray ionization
*GG*: *Glycyrrhiza glabra*
HCD: Higher-energy collision dissociation
HPLC: HIGH performance liquid chromatography
ICAM-1: Intracellular adhesion molecule-1
ILβ4: Interleukinβ-4
LC/MS/MS: Liquid chromatography–electrospray ionization–tandem mass spectrometry
LTB4: Leukotriene B4
MS: Mass spectrometry
QC: Quality control
UPLC-HRMS: Ultra-performance liquid chromatography-electrospray high resolution mass spectrometry
